# Supplementary material for: Heterologous vaccination regimens with self-amplifying RNA and adenoviral COVID vaccines induce robust immune responses in mice
Source: Nat Commun. 2021 May 17;12:2893. doi: 10.1038/s41467-021-23173-1 (PMC8129084; doi:10.1038/s41467-021-23173-1)
Supplement: Supplementary file 1 — Supplementary Information [file 41467_2021_23173_MOESM1_ESM.pdf]

Supplementary Figures

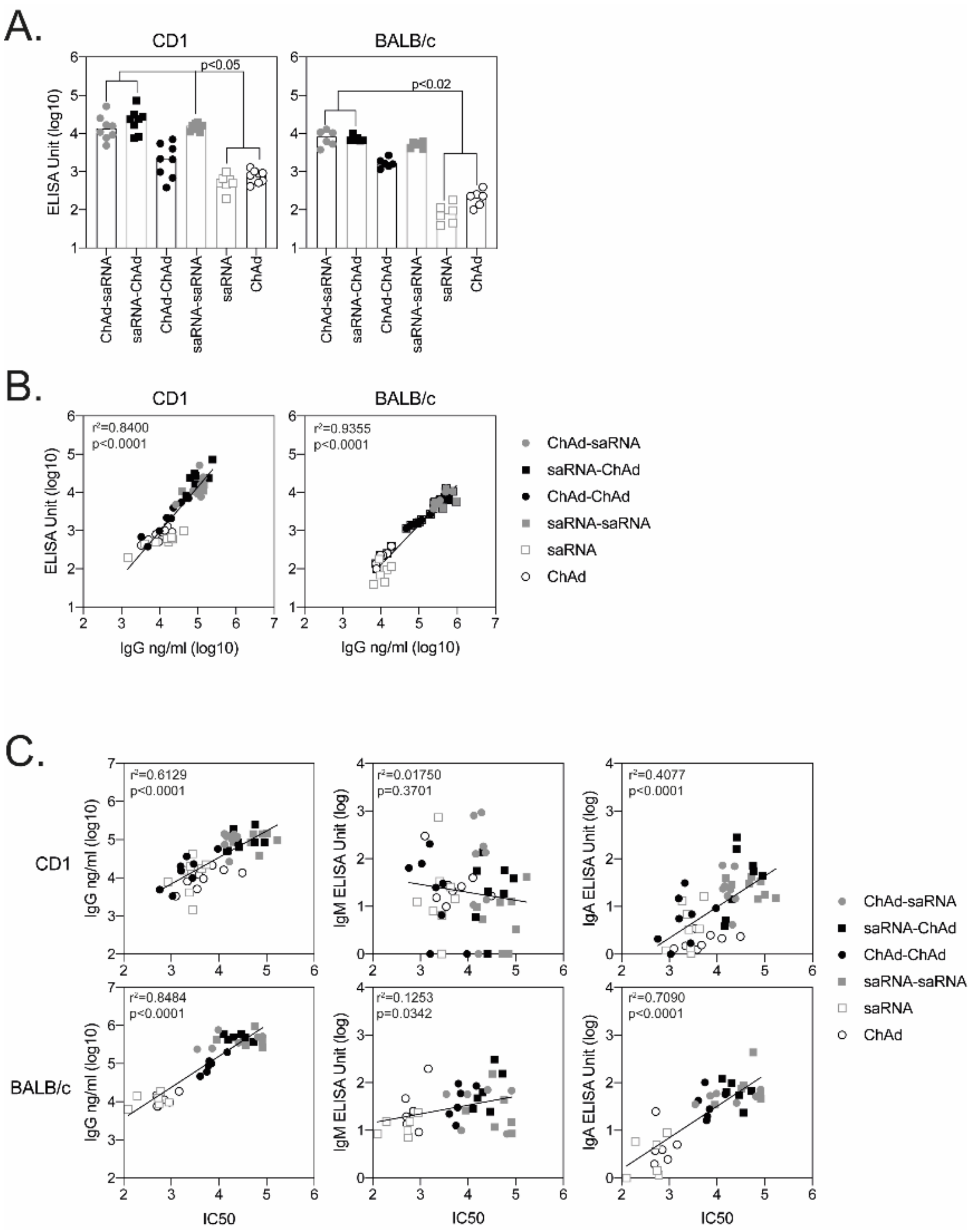


**Fig S1: Comparison of assays measuring antibody responses after vaccination**
SARS-CoV-2 spike-specific IgG responses were measured in the serum of CD1 (n=8) and BALB/c (n=6) mice collected 3 weeks after the final immunisation in a standardised ELISA (**A.**) and compared to IgG responses measured as a concentration (**B.**).
**C.**) Graphs show relationship between IgG, IgM and IgA responses to SARS-CoV-2 pseudoneutralisation IC50 values presented by linear
regression on log-transformed values. Data points represent individual animals from all groups together (CD1 n=8, BALB/c n=6), $r^2$ and significant p values ($p<0.05$) are indicated on each graph.

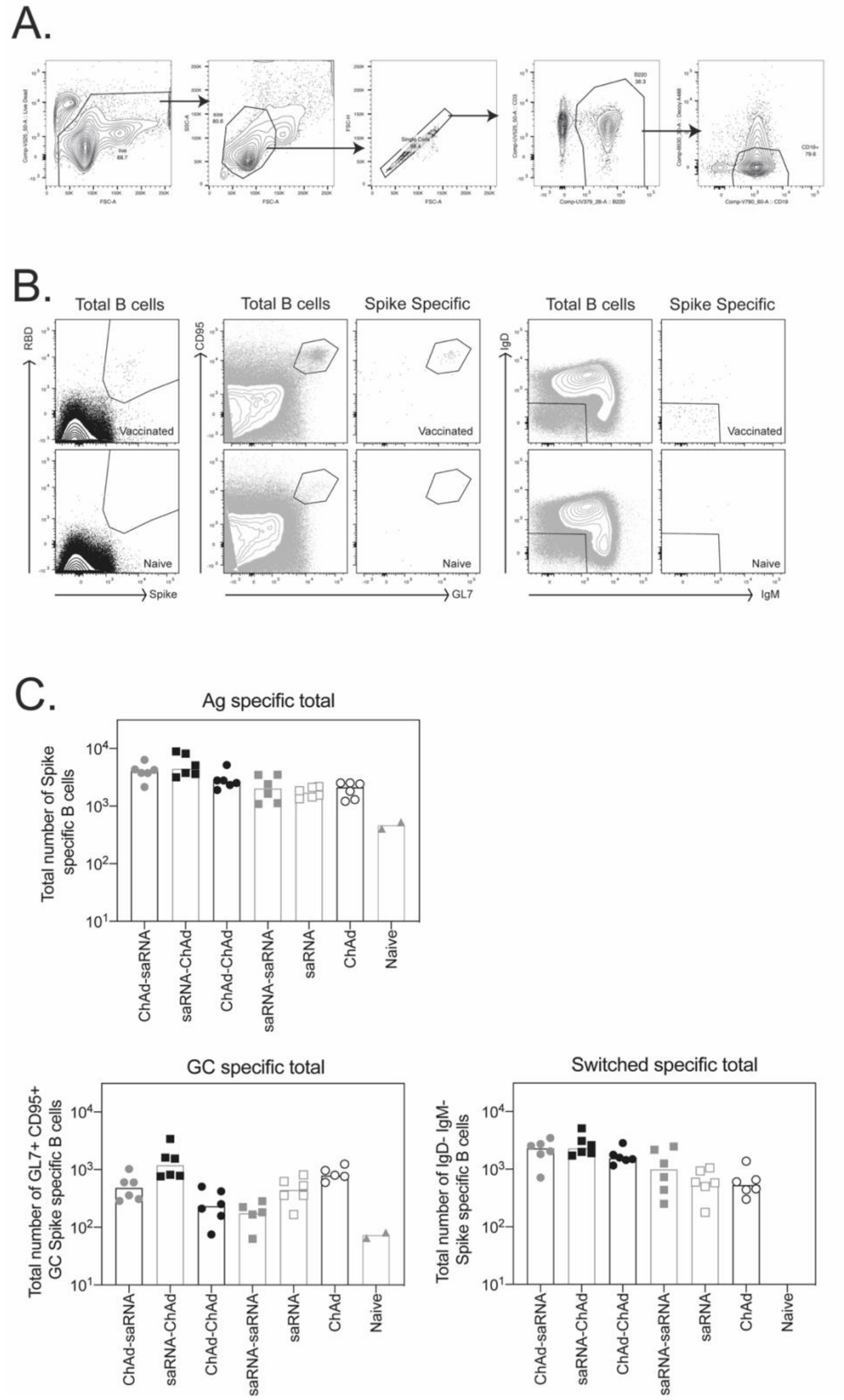


**Fig S2: Antigen specific B cell responses**

SARS-CoV-2 spike-specific B cells responses were measured in the spleen of BALB/c mice (n=6) 3 weeks after the final immunisation. **A.**) Plots show the gating strategy for identification of B cells. **B.**) Antigen specific cells were identified by positive binding to spike-PE and RBD-A647 tetramers. Germinal center (GC) B cells were identified as GL7$^+$ and CD95$^+$, switched B cells identified as IgD$^-$ and IgM$^-$ antigen-specific B cells **C.**) Graphs show the total number of antigen-specific B cells, antigen-specific GC B cells and antigen-specific switched B cells, data points are representative of individual mice, median responses per group indicated by bars.

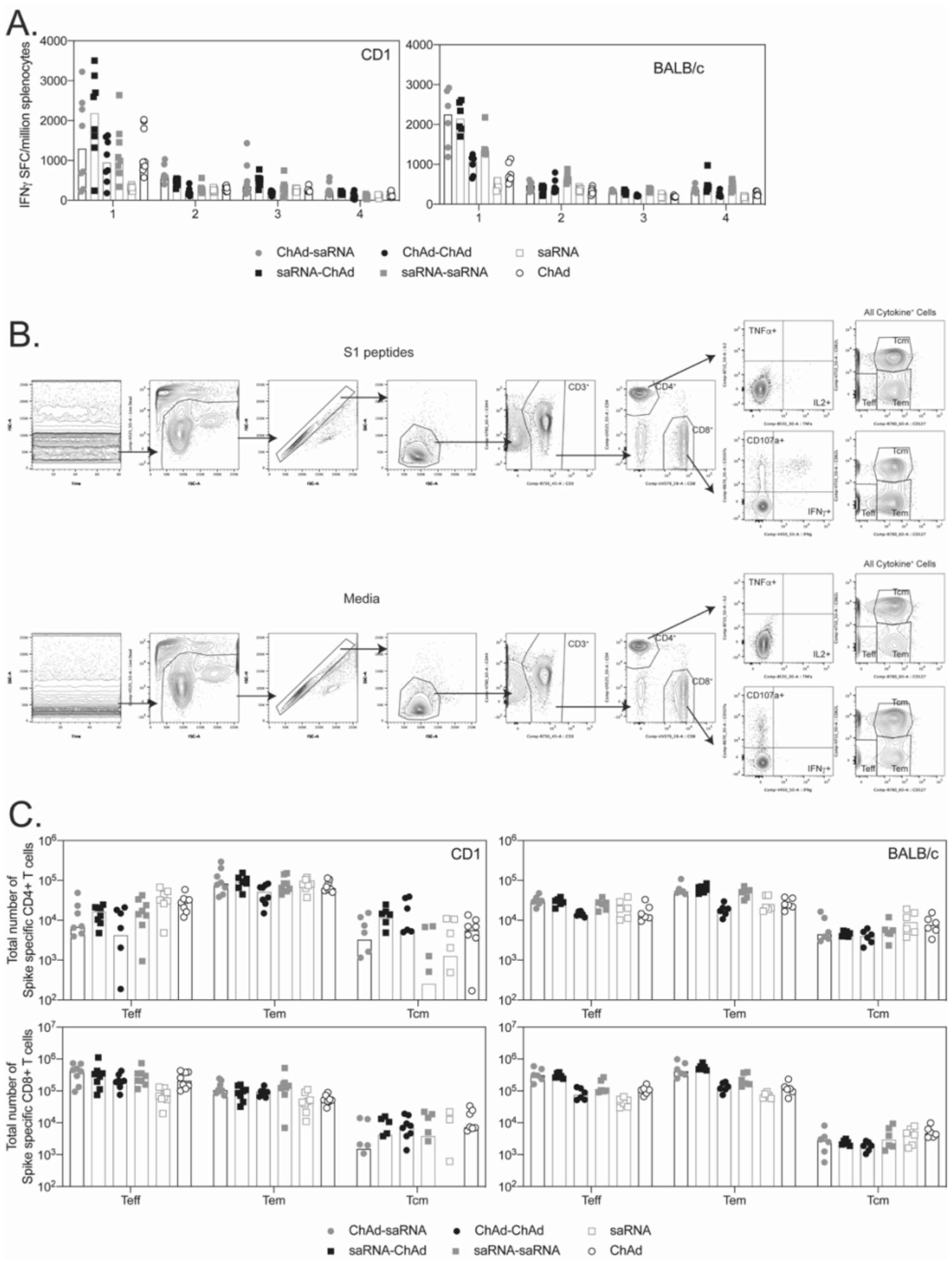


**Fig S3: T cell responses measured by ELISpot and ICS**

**A.**) Graphs show the IFNγ SFC detected by IFNγ ELISpot to each individual pool of peptides covering the length of SARs CoV2 spike protein in the spleens of CD1 (n=8) and BALB/c (n=6) mice collected 3 weeks after the final immunisation. Each data point represents an individual mouse, bars represent the median response per group.

**B.**) Plots show the gating strategy used to identify antigen specific T cell responses.

**C.**) Graphs show the total number of Teff, Tem and Tcm responses detected in the spleens of CD1 (n=8) and BALB/c (n=6) mice collected 3 weeks after the final immunisation. Each point indicates an individual mouse, bars represent the median response per group.

## **Table S1:** Overlapping peptides spanning the length of SARs CoV2 spike protein

| **S1 region** | | | | **S2 region** | | | |
|---|---|---|---|---|---|---|---|
| **Pool 1** | | **Pool 2** | | **Pool 3** | | **Pool 4** | |
| **#** | **Sequence** | **#** | **Sequence** | **#** | **Sequence** | **#** | **Sequence** |
| 1 | MFVFLVLLPLVSSQC | 78 | EKGIYQTSNFRVQPT | 168 | GICASYQTQTNSPRR | 242 | QLSSNFGAISSVLND |
| 2 | LVLLPLVSSQCVNLT | 79 | YQTSNFRVQPTESIV | 169 | SYQTQTNSPRRARSV | 243 | NFGAISSVLNDILSR |
| 3 | PLVSSQCVNLTTRTQ | 80 | NFRVQPTESIVRFPN | 170 | QTNSPRRARSVASQS | 244 | ISSVLNDILSRLDKV |
| 4 | SQCVNLTTRTQLPPA | 81 | QPTESIVRFPNITNL | 171 | PRRARSVASQSIIAY | 245 | LNDILSRLDKVEAEV |
| 5 | NLTTRTQLPPAYTNS | 82 | SIVRFPNITNLCPFG | 172 | RSVASQSIIAYTMSL | 246 | LSRLDKVEAEVQIDR |
| 6 | RTQLPPAYTNSFTRG | 83 | FPNITNLCPFGEVFN | 173 | SQSIIAYTMSLGAEN | 247 | DKVEAEVQIDRLITG |
| 7 | PPAYTNSFTRGVYYP | 84 | TNLCPFGEVFNATRF | 174 | IAYTMSLGAENSVAY | 248 | AEVQIDRLITGRLQS |
| 8 | TNSFTRGVYYPDKVF | 85 | PFGEVFNATRFASVY | 175 | MSLGAENSVAYSNNS | 249 | IDRLITGRLQSLQTY |
| 9 | TRGVYYPDKVFRSSV | 86 | VFNATRFASVYAWNR | 176 | AENSVAYSNNSIAIP | 250 | ITGRLQSLQTYVTQQ |
| 10 | YYPDKVFRSSVLHST | 87 | TRFASVYAWNRKRIS | 177 | VAYSNNSIAIPTNFT | 251 | LQSLQTYVTQQLIRA |
| 11 | KVFRSSVLHSTQDLF | 88 | SVYAWNRKRISNCVA | 178 | NNSIAIPTNFTISVT | 252 | QTYVTQQLIRAAEIR |
| 12 | SSVLHSTQDLFLPFF | 89 | WNRKRISNCVADYSV | 179 | AIPTNFTISVTTEIL | 253 | TQQLIRAAEIRASAN |
| 13 | HSTQDLFLPFFSNVT | 90 | RISNCVADYSVLYNS | 180 | NFTISVTTEILPVSM | 254 | IRAAEIRASANLAAT |
| 14 | DLFLPFFSNVTWFHA | 91 | CVADYSVLYNSASFS | 181 | SVTTEILPVSMTKTS | 255 | EIRASANLAATKMSE |
| 15 | PFFSNVTWFHAIHVS | 92 | YSVLYNSASFSTFKC | 182 | EILPVSMTKTSVDCT | 256 | SANLAATKMSECVLG |
| 16 | NVTWFHAIHVSGTNG | 93 | YNSASFSTFKCYGVS | 183 | VSMTKTSVDCTMYIC | 257 | AATKMSECVLGQSKR |
| 17 | FHAIHVSGTNGTKRF | 94 | SFSTFKCYGVSPTKL | 184 | KTSVDCTMYICGDST | 258 | MSECVLGQSKRVDFC |
| 18 | HVSGTNGTKRFDNPV | 95 | FKCYGVSPTKLNDLC | 185 | DCTMYICGDSTECSN | 259 | VLGQSKRVDFCGKGY |
| 19 | TNGTKRFDNPVLPFN | 96 | GVSPTKLNDLCFTNV | 186 | YICGDSTECSNLLLQ | 260 | SKRVDFCGKGYHLMS |
| 20 | KRFDNPVLPFNDGVY | 97 | TKLNDLCFTNVYADS | 187 | DSTECSNLLLQYGSF | 261 | DFCGKGYHLMSFPQS |
| 21 | NPVLPFNDGVYFAST | 98 | DLCFTNVYADSFVIR | 188 | CSNLLLQYGSFCTQL | 262 | KGYHLMSFPQSAPHG |
| 22 | PFNDGVYFASTEKSN | 99 | TNVYADSFVIRGDEV | 189 | LLQYGSFCTQLNRAL | 263 | LMSFPQSAPHGVVFL |
| 23 | GVYFASTEKSNIIRG | 100 | ADSFVIRGDEVRQIA | 190 | GSFCTQLNRALTGIA | 264 | PQSAPHGVVFLHVTY |
| 24 | ASTEKSNIIRGWIFG | 101 | VIRGDEVRQIAPGQT | 191 | TQLNRALTGIAVEQD | 265 | PHGVVFLHVTYVPAQ |
| 25 | KSNIIRGWIFGTTLD | 102 | DEVRQIAPGQTGKIA | 192 | RALTGIAVEQDKNTQ | 266 | VFLHVTYVPAQEKNF |
| 26 | IRGWIFGTTLDSKTQ | 103 | QIAPGQTGKIADYNY | 193 | GIAVEQDKNTQEVFA | 267 | VTYVPAQEKNFTTAP |
| 27 | IFGTTLDSKTQSLLI | 104 | GQTGKIADYNYKLPD | 194 | EQDKNTQEVFAQVKQ | 268 | PAQEKNFTTAPAICH |
| 28 | TLDSKTQSLLIVNNA | 105 | KIADYNYKLPDDFTG | 195 | NTQEVFAQVKQIYKT | 269 | KNFTTAPAICHDGKA |
| 29 | KTQSLLIVNNATNVV | 106 | YNYKLPDDFTGCVIA | 196 | VFAQVKQIYKTPPIK | 270 | TAPAICHDGKAHFPR |
| 30 | LLIVNNATNVVIKVC | 107 | LPDDFTGCVIAWNSN | 197 | VKQIYKTPPIKDFGG | 271 | ICHDGKAHFPREGVF |
| 31 | NNATNVVIKVCEFQF | 108 | FTGCVIAWNSNNLDS | 198 | YKTPPIKDFGGFNFS | 272 | GKAHFPREGVFVSNG |
| 32 | NVVIKVCEFQFCNDP | 109 | VIAWNSNNLDSKVGG | 199 | PIKDFGGFNFSQILP | 273 | FPREGVFVSNGTHWF |
| 33 | KVCEFQFCNDPFLGV | 110 | NSNNLDSKVGGNYNY | 200 | FGGFNFSQILPDPSK | 274 | GVFVSNGTHWFVTQR |
| 34 | FQFCNDPFLGVYYHK | 111 | LDSKVGGNYNYLYRL | 201 | NFSQILPDPSKPSKR | 275 | SNGTHWFVTQRNFYE |
| 35 | NDPFLGVYYHKNNKS | 112 | VGGNYNYLYRLFRKS | 202 | ILPDPSKPSKRSFIE | 276 | HWFVTQRNFYEPQII |
| 36 | LGVYYHKNNKSWMES | 113 | YNYLYRLFRKSNLKP | 203 | PSKPSKRSFIEDLLF | 277 | TQRNFYEPQIITTDN |
| 37 | YHKNNKSWMESEFRV | 114 | YRLFRKSNLKPFERD | 204 | SKRSFIEDLLFNKVT | 278 | FYEPQIITTDNTFVS |
| 38 | NKSWMESEFRVYSSA | 115 | RKSNLKPFERDISTE | 205 | FIEDLLFNKVTLADA | 279 | QIITTDNTFVSGNCD |
| 39 | MESEFRVYSSANNCT | 116 | LKPFERDISTEIYQA | 206 | LLFNKVTLADAGFIK | 280 | TDNTFVSGNCDVVIG |
| 40 | FRVYSSANNCTFEYV | 117 | ERDISTEIYQAGSTP | 207 | KVTLADAGFIKQYGD | 281 | FVSGNCDVVIGIVNN |
| 41 | SSANNCTFEYVSQPF | 118 | STEIYQAGSTPCNGV | 208 | ADAGFIKQYGDCLGD | 282 | NCDVVIGIVNNTVYD |
| 42 | NCTFEYVSQPFLMDL | 119 | YQAGSTPCNGVEGFN | 209 | FIKQYGDCLGDIAAR | 283 | VIGIVNNTVYDPLQP |
| 43 | EYVSQPFLMDLEGKQ | 120 | STPCNGVEGFNCYFP | 210 | YGDCLGDIAARDLIC | 284 | VNNTVYDPLQPELDS |
| 44 | QPFLMDLEGKQGNFK | 121 | NGVEGFNCYFPLQSY | 211 | LGDIAARDLICAQKF | 285 | VYDPLQPELDSFKEE |
| 45 | MDLEGKQGNFKNLRE | 122 | GFNCYFPLQSYGFQP | 212 | AARDLICAQKFNGLT | 286 | LQPELDSFKEELDKY |
| 46 | GKQGNFKNLREFVFK | 123 | YFPLQSYGFQPTNGV | 213 | LICAQKFNGLTVLPP | 287 | LDSFKEELDKYFKNH |
| 47 | NFKNLREFVFKNIDG | 124 | QSYGFQPTNGVGYQP | 214 | QKFNGLTVLPPLLTD | 288 | KEELDKYFKNHTSPD |
| 48 | LREFVFKNIDGYFKI | 125 | FQPTNGVGYQPYRVV | 215 | GLTVLPPLLTDEMIA | 289 | DKYFKNHTSPDVDLG |
| 49 | VFKNIDGYFKIYSKH | 126 | NGVGYQPYRVVVLSF | 216 | LPPLLTDEMIAQYTS | 290 | KNHTSPDVDLGDISG |
| 50 | IDGYFKIYSKHTPIN | 127 | YQPYRVVVLSFELLH | 217 | LTDEMIAQYTSALLA | 291 | SPDVDLGDISGINAS |
| 51 | FKIYSKHTPINLVRD | 128 | RVVVLSFELLHAPAT | 218 | MIAQYTSALLAGTIT | 292 | DLGDISGINASVVNI |
| 52 | SKHTPINLVRDLPQG | 129 | LSFELLHAPATVCGP | 219 | YTSALLAGTITSGWT | 293 | ISGINASVVNIQKEI |
| 53 | PINLVRDLPQGFSAL | 130 | LLHAPATVCGPKKST | 220 | LLAGTITSGWTFGAG | 294 | NASVVNIQKEIDRLN |
| 54 | VRDLPQGFSALEPLV | 131 | PATVCGPKKSTNLVK | 221 | TITSGWTFGAGAALQ | 295 | VNIQKEIDRLNEVAK |
| 55 | PQGFSALEPLVDLPI | 132 | CGPKKSTNLVKNKCV | 222 | GWTFGAGAALQIPFA | 296 | KEIDRLNEVAKNLNE |
| 56 | SALEPLVDLPIGINI | 133 | KSTNLVKNKCVNFNF | 223 | GAGAALQIPFAMQMA | 297 | RLNEVAKNLNESLID |
| 57 | PLVDLPIGINITRFQ | 134 | LVKNKCVNFNFNGLT | 224 | ALQIPFAMQMAYRFN | 298 | VAKNLNESLIDLQEL |
| 58 | LPIGINITRFQTLLA | 135 | KCVNFNFNGLTGTGV | 225 | PFAMQMAYRFNGIGV | 299 | LNESLIDLQELGKYE |
| 59 | INITRFQTLLALHRS | 136 | FNFNGLTGTGVLTES | 226 | QMAYRFNGIGVTQNV | 300 | LIDLQELGKYEQYIK |
| 60 | RFQTLLALHRSYLTP | 137 | GLTGTGVLTESNKKF | 227 | RFNGIGVTQNVLYEN | 301 | QELGKYEQYIKWPWY |
| 61 | LLALHRSYLTPGDSS | 138 | TGVLTESNKKFLPFQ | 228 | IGVTQNVLYENQKLI | 302 | KYEQYIKWPWYIWLG |
| 62 | HRSYLTPGDSSSGWT | 139 | TESNKKFLPFQQFGR | 229 | QNVLYENQKLIANQF | 303 | YIKWPWYIWLGFIAG |
| 63 | LTPGDSSSGWTAGAA | 140 | KKFLPFQQFGRDIAD | 230 | YENQKLIANQFNSAI | 304 | PWYIWLGFIAGLIAI |
| 64 | DSSSGWTAGAAAYYV | 141 | PFQQFGRDIADTTDA | 231 | KLIANQFNSAIGKIQ | 305 | WLGFIAGLIAIVMVT |
| 65 | GWTAGAAAYYVGYLQ | 142 | FGRDIADTTDAVRDP | 232 | NQFNSAIGKIQDSLS | 306 | IAGLIAIVMVTIMLC |
| 66 | GAAAYYVGYLQPRTF | 143 | IADTTDAVRDPQTLE | 233 | SAIGKIQDSLSSTAS | 307 | IAIVMVTIMLCCMTS |
| 67 | YYVGYLQPRTFLLKY | 144 | TDAVRDPQTLEILDI | 234 | KIQDSLSSTASALGK | 308 | MVTIMLCCMTSCCSC |
| 68 | YLQPRTFLLKYNENG | 145 | RDPQTLEILDITPCS | 235 | SLSSTASALGKLQDV | 309 | MLCCMTSCCSCLKGC |
| 69 | RTFLLKYNENGTITD | 146 | TLEILDITPCSFGGV | 236 | TASALGKLQDVVNQN | 310 | MTSCCSCLKGCCSCG |
| 70 | LKYNENGTITDAVDC | 147 | LDITPCSFGGVSVIT | 237 | LGKLQDVVNQNAQAL | 311 | CSCLKGCCSCGSCCK |
| 71 | ENGTITDAVDCALDP | 148 | PCSFGGVSVITPGTN | 238 | QDVVNQNAQALNTLV | 312 | KGCCSCGSCCKFDED |
| 72 | ITDAVDCALDPLSET | 149 | GGVSVITPGTNTSNQ | 239 | NQNAQALNTLVKQLS | 313 | SCGSCCKFDEDDSEP |
| 73 | VDCALDPLSETKCTL | 150 | VITPGTNTSNQVAVL | 240 | QALNTLVKQLSSNFG | 314 | CCKFDEDDSEPVLKG |

| | | | | | | | |
|---|---|---|---|---|---|---|---|
| 74 | LDPLSETKCTLKSFT | 151 | GTNTSNQVAVLYQDV | 241 | TLVKQLSSNFGAISS | 315 | DEDDSEPVLKGVKLH |
| 75 | SETKCTLKSFTVEKG | 152 | SNQVAVLYQDVNCTE | | | 316 | DDSEPVLKGVKLHYT |
| 76 | CTLKSFTVEKGIYQT | 153 | AVLYQDVNCTEVPVA | | | | |
| 77 | SFTVEKGIYQTSNFR | 154 | QDVNCTEVPVAIHAD | | | | |
| | | 155 | CTEVPVAIHADQLTP | | | | |
| tpa | MDAMKRGLCCVLLLC | 156 | PVAIHADQLTPTWRV | | | | |
| tpa | RGLCCVLLLCGAVFV | 157 | HADQLTPTWRVYSTG | | | | |
| tpa | VLLLCGAVFVSASQE | 158 | LTPTWRVYSTGSNVF | | | | |
| tpa | GAVFVSASQEIHARF | 159 | WRVYSTGSNVFQTRA | | | | |
| tpa | SASQEIHARFRRIHS | 160 | STGSNVFQTRAGCLI | | | | |
| | | 161 | NVFQTRAGCLIGAEH | | | | |
| | | 162 | TRAGCLIGAEHVNNS | | | | |
| | | 163 | CLIGAEHVNNSYECD | | | | |
| | | 164 | AEHVNNSYECDIPIG | | | | |
| | | 165 | NNSYECDIPIGAGIC | | | | |
| | | 166 | ECDIPIGAGICASYQ | | | | |
| | | 167 | PIGAGICASYQTQTN | | | | |
