# Supplementary material for: Heterologous vaccination regimens with self-amplifying RNA and adenoviral COVID vaccines induce robust immune responses in mice
Source: Nat Commun. 2021 May 17;12:2893. doi: 10.1038/s41467-021-23173-1 (PMC8129084; doi:10.1038/s41467-021-23173-1)
Supplement: Supplementary file 3 — Reporting Summary [file 41467_2021_23173_MOESM3_ESM.pdf]

Corresponding author(s): Alexandra J Spencer

Last updated by author(s): 13th April 2021

Reporting Summary

Nature Research wishes to improve the reproducibility of the work that we publish. This form provides structure for consistency and transparency in reporting. For further information on Nature Research policies, see our Editorial Policies and the Editorial Policy Checklist.

Statistics

For all statistical analyses, confirm that the following items are present in the figure legend, table legend, main text, or Methods section.

n/a	Confirmed	
☐	☒	The exact sample size (n) for each experimental group/condition, given as a discrete number and unit of measurement
☐	☒	A statement on whether measurements were taken from distinct samples or whether the same sample was measured repeatedly
☐	☒	The statistical test(s) used AND whether they are one- or two-sided<br>*Only common tests should be described solely by name; describe more complex techniques in the Methods section.*
☐	☒	A description of all covariates tested
☐	☒	A description of any assumptions or corrections, such as tests of normality and adjustment for multiple comparisons
☐	☒	A full description of the statistical parameters including central tendency (e.g. means) or other basic estimates (e.g. regression coefficient) AND variation (e.g. standard deviation) or associated estimates of uncertainty (e.g. confidence intervals)
☐	☒	For null hypothesis testing, the test statistic (e.g. F, t, r) with confidence intervals, effect sizes, degrees of freedom and P value noted<br>*Give P values as exact values whenever suitable.*
☒	☐	For Bayesian analysis, information on the choice of priors and Markov chain Monte Carlo settings
☒	☐	For hierarchical and complex designs, identification of the appropriate level for tests and full reporting of outcomes
☒	☐	Estimates of effect sizes (e.g. Cohen's d, Pearson's r), indicating how they were calculated

Our web collection on statistics for biologists contains articles on many of the points above.

Software and code

Policy information about availability of computer code

Data collection	Flow cytometry data was acquired on FACsDiva Software, ELISA data was acquired through SOFTmax PRO software, ELISPot data acquired by counting spots using AID ELISpot Software
Data analysis	GraphPad Prism v8 and v9; Excel; FlowJo v10, SOFTmax PRO software, AID ELISpot Software

For manuscripts utilizing custom algorithms or software that are central to the research but not yet described in published literature, software must be made available to editors and reviewers. We strongly encourage code deposition in a community repository (e.g. GitHub). See the Nature Research guidelines for submitting code & software for further information.

Data

Policy information about availability of data

All manuscripts must include a data availability statement. This statement should provide the following information, where applicable:

- Accession codes, unique identifiers, or web links for publicly available datasets
- A list of figures that have associated raw data
- A description of any restrictions on data availability

All data available with the manuscript and a source data excel file is supplied with the manuscript

Field-specific reporting

Life sciences study design

All studies must disclose on these points even when the disclosure is negative.

Sample size	Sample size used was based on a power calculation utilizing prior immunogenicity data from these strains of mice.
Data exclusions	No data was excluded
Replication	Experiment was performed 3 times, in 2 different strains of mice to confirm the effect, the data was consistent across all experiments.
Randomization	Mice were randomly allocated into groups on arrival in the facility.
Blinding	Blinding was not applied, due to limited number of staff available to assist with these studies. Where feasible, staff were blinded to groupings until after generation of the raw data.

Reporting for specific materials, systems and methods

We require information from authors about some types of materials, experimental systems and methods used in many studies. Here, indicate whether each material, system or method listed is relevant to your study. If you are not sure if a list item applies to your research, read the appropriate section before selecting a response.

Materials & experimental systems

n/a	Involved in the study	
☐	☒	Antibodies
☐	☒	Eukaryotic cell lines
☒	☐	Palaeontology and archaeology
☐	☒	Animals and other organisms
☒	☐	Human research participants
☒	☐	Clinical data
☒	☐	Dual use research of concern

Methods

n/a	Involved in the study	
☒	☐	ChIP-seq
☐	☒	Flow cytometry
☒	☐	MRI-based neuroimaging

Antibodies

Antibodies used

Flow cytometry antibodies included anti-mouse TNFa Alexa 488 (Biolegend, Cat Number:506313 Lot Number :B253791), anti-mouse IL2 PerCPCy5.5 (Biolegend, Cat Number:503822 Lot Number :B282323), anti-mouse IFNg e450 (Thermofisher, Cat Number:48-7311-82 Lot Number :E10946-1634), anti-mouse IL4 BV605 (Biolegend, Cat Number:504126 Lot Number :B301449), anti-mouse CD62L BV711 (Biolegend, Cat Number:104445 Lot Number :B291923), anti-mouse CD44 BV780 (Biolegend, Cat Number:103059 Lot Number :B281255), anti-mouse CD107a Alexa647 (Biolegend, Cat Number:121610 Lot Number :B254174), anti-mouse CD3 Alexa700 (Biolegend, Cat Number:100216 Lot Number :B263796), anti-mouse CD127 APCCy7 (Biolegend, Cat Number:135040 Lot Number :B283381), anti-mouse IL10 PE (BD, Cat Number:554467 Lot Number :B249530), anti-mouse CD69 PECy7 (Biolegend, Cat Number:104512 Lot Number :B291439), anti-mouse CD8 BUV395 (BD, Cat Number:563786 Lot Number :91417), anti-mouse CD4 BUV496 (BD, Cat Number:564667 Lot Number :205396), anti-PfCSP NANP9-Alexa488 (in house), anti-mouse GL7 PerCPCy5.5 (Biolegend, Cat Number:144609 Lot Number :B306885), anti-mouse CD138 Pacific Blue (BD, Cat Number:553714 Lot Number :B260840), anti-mouse CD95 BV605 (Biolegend, Cat Number:152612 Lot Number :B285270), anti-mouse CD4 BV650 (Biolegend, Cat Number:100469 Lot Number :B258780), anti-mouse CD279 BV711 (Biolegend, Cat Number:135231 Lot Number :B284699), anti-mouse CD19 BV780 (Biolegend, Cat Number:115543 Lot Number :B270691), RBD-A647 (in house), anti-mouse IgD Alexa700 (Biolegend, Cat Number:405730 Lot Number :B255982), anti-mouse IgM APC-eFluro780 (Thermofisher, Cat Number:47-5790-82 Lot Number :), Spike-PE (in house), anti-mouse CD38 PECy5 (Thermofisher, Cat Number:15-0381-82 Lot Number :15-0381-82), anti-mouse CD69 PECy7 (Biolegend, Cat Number:104512 Lot Number :B291439), anti-mouse CD45R (B220) BUV395 (BD, Cat Number:563793 Lot Number :9204546), anti-mouse CD3 BUV496 (BD, Cat Number:564661 Lot Number :198383), Fc Block (anti-mouse CD16/CD32) (Thermofisher, Cat Number:14-016186 Lot Number :E03519-1635), live dead Aqua (thermofisher, Cat Number: Lot Number :2204201).

ELISA antibodies included anti-mouse IgG (Sigma-Aldrich, Cat No:A3562, Lot No:SLBK6489V, Dilution:1 in 5000), anti-mouse IgM (Abcam, Cat No:ab98672, Lot No:GR3304914-1, Dilution:1 in 5000), anti-mouse IgA (Southern Biotech, Cat No:1040-04, Lot No:J4416-QI18F, Dilution:1 in 1000), anti-mouse IgG1 (Southern Biotech, Cat No:1071-04, Lot No:B5312-N140, Dilution:1 in 4000), anti-mouse IgG2a (Southern Biotech, Cat No:1081-04, Lot No:J0216-V459B, Dilution:1 in 4000), anti-mouse IgG2b (Southern Biotech, Cat No:1091-04, Lot No:J1111-SA69, Dilution:1 in 4000), anti-mouse IgG2c (Southern Biotech, Cat No:1078-04, Lot No:L3913-NA60B, Dilution:1 in 4000), anti-mouse IgG3 (Abcam, Cat No:ab98705, Lot No:GR3211365-1, Dilution:1 in 1000), anti-mouse Kappa (Serotech, Cat No:1050-01 , Lot No:A3520-XK60, Dilution:1 in 1000), anti-mouse lambda (Serotech, Cat No:1060-01, Lot No:L0612-WC60, Dilution:1 in 1000), Anti-mouse IgG HRP (Serotech, Cat No:1030-05, Lot No:E2518-V099C, Dilution:1 in 2000).

Validation

All antibodies were validated for use with mouse samples by the supplier with validation information available on their relevant websites.

Flow cytometry antibodies:

Biolegend: each lot of antibody is quality control tested in intracellular immunofluorescent staining with flow cytometric analysis.
Thermofisher: each lot of antibody is verified by cell treatment to ensure that the antibody binds to the antigen stated.
BD Bioscience: each antibody is tested and confirmed for use in multicolor flow cytometric analyses.
Antibodies are titrated in house to optimise staining panels.

ELISA antibodies:
Sigma Aldrich: Anti-Mouse IgG (whole molecule)-Alkaline Phosphatase antibody is suitable for use in direct ELISA and western blot.
Abcam: Our Abpromise guarantee covers the use of ab98672 and ab98705 for use in ELISA.
Serotech (now BioRad): This product has been reported to work in the following applications (ELISA, Flow Cytometry, Immunoprecipitation). This information is derived from testing within our laboratories, peer-reviewed publications or personal communications from the originators. Please refer to references indicated for further information. For general protocol recommendations, please visit the antibody protocols page.
Serotech: Quality tested applications for relevant formats include - ELISA, FLISA

Eukaryotic cell lines

Policy information about cell lines

Cell line source(s)	HEK293T.17 cells were purchased from ATCC
Authentication	Cell lines were authenticated by supplier
Mycoplasma contamination	All cell lines routinely tested negative for mycoplasma contamination
Commonly misidentified lines (See ICLAC register)	N/A

Animals and other organisms

Policy information about studies involving animals; ARRIVE guidelines recommended for reporting animal research

Laboratory animals	Inbred female BALB/cOlaHsd (BALB/c) and outbred Crl:CD1 (CD1) mice were purchase from commercial suppliers (Envigo and Charles River Laboratories or Envigo) and randomly allocated into ‘prime-only’ or ‘prime-boost’ vaccination groups (BALB/c n=6 and CD1 n=8) upon arrival. Mice were 7 weeks of age at the start of the experiment.
Wild animals	No wild animals were used in this study
Field-collected samples	No field collected samples were used in this study
Ethics oversight	Studies were performed in accordance with the UK Animals (Scientific Procedures) Act 1986 and with approval from the relevant local Animal Welfare and Ethical Review Body (AWERB) (Mice - Project License P9808B4F1 and University of Oxford AWERB).

Note that full information on the approval of the study protocol must also be provided in the manuscript.

Flow Cytometry

Plots

Confirm that:

- [x] The axis labels state the marker and fluorochrome used (e.g. CD4-FITC).
- [x] The axis scales are clearly visible. Include numbers along axes only for bottom left plot of group (a 'group' is an analysis of identical markers).
- [x] All plots are contour plots with outliers or pseudocolor plots.
- [x] A numerical value for number of cells or percentage (with statistics) is provided.

Methodology

Sample preparation	Sample preparation: Single cell suspension of murine splenocytes were prepared by passing cells through 70μM cell strainers and ACK lysis prior to resuspension in complete medium. Cells were stimulated at 37oC for 6 hours with 2μg/ml S1 or S2 pools of peptide, media or cell stimulation cocktail (containing PMA-Ionomycin, Biolegend), together with 1μg/ml Golgi-plug (BD) with the addition of 2μl/ml CD107a-Alexa647).
Instrument	BD FortessaX2
Software	FACS DIVA software
Cell population abundance	For mouse samples, an an acquisition threshold was set at a minimum of 5000 events in the live CD3+ gate
Gating strategy	For identification of antigen specific B cells Antigen specific B cells were identified by gating on LIVE/DEAD negative, size (FSC-A vs SSC), doublet negative (FSC-H vs FSC-A), CD45RA+, CD19+ and NANP-A488-, RBD-A647+ and Spike-PE+ .

Antigen specific T cells were identified by gating on LIVE/DEAD negative, doublet negative (FSC-H vs FSC-A), size (FSC-A vs SSC), CD3+, CD4+ or CD8+ cells.

☒ Tick this box to confirm that a figure exemplifying the gating strategy is provided in the Supplementary Information.
